# From DNA-protein interactions to the genetic circuit design using CRISPR-dCas systems

**DOI:** 10.3389/fmolb.2022.1070526

**Published:** 2022-12-14

**Authors:** A. K. Shaytan, R. V. Novikov, R. S. Vinnikov, A. K. Gribkova, G. S. Glukhov

**Affiliations:** ^1^ Department of Biology, Lomonosov Moscow State University, Moscow, Russia; ^2^ Department of Computer Science, HSE University, Moscow, Russia; ^3^ Department of Bioengineering and Bioinformatics, Lomonosov Moscow State University, Moscow, Russia; ^4^ Faculty of Biology, MSU-BIT Shenzhen University, Shenzhen, China

**Keywords:** synthetic genetic circuits, CRISPR-dCas, dCas, CRISPRi, CRISPRa, transcription

## Abstract

In the last decade, the CRISPR-Cas technology has gained widespread popularity in different fields from genome editing and detecting specific DNA/RNA sequences to gene expression control. At the heart of this technology is the ability of CRISPR-Cas complexes to be programmed for targeting particular DNA loci, even when using catalytically inactive dCas-proteins. The repertoire of naturally derived and engineered dCas-proteins including fusion proteins presents a promising toolbox that can be used to construct functional synthetic genetic circuits. Rational genetic circuit design, apart from having practical relevance, is an important step towards a deeper quantitative understanding of the basic principles governing gene expression regulation and functioning of living organisms. In this minireview, we provide a succinct overview of the application of CRISPR-dCas-based systems in the emerging field of synthetic genetic circuit design. We discuss the diversity of dCas-based tools, their properties, and their application in different types of genetic circuits and outline challenges and further research directions in the field.

## 1 Introduction

Interactions of proteins and nucleic acids are the cornerstone of genome functioning in living organisms. They mediate the execution of complex genetic and epigenetic networks and circuits in bacteria, archaea, eukaryotes, and viruses. Understanding the molecular mechanisms behind living organisms’ operation now goes hand-in-hand with the advances in synthetic biology which aims to construct biological systems and their components in a bottom-up approach (Jia and Schwille, 2019). A quote by Nobel laureate Richard Feynman “What I cannot create, I do not understand” nicely illustrates this trend and the challenges ahead.

Around 20 years ago two pioneering works by Elowitz and Leibler (2000) and Gardner et al. (2000) showcased the first artificial transcriptional genetic circuits in bacteria. These circuits implemented periodic oscillations of gene expression (repressilator circuit) and persistent switching between the expression of two genes upon transient exposure to a chemical signal (toggle switch circuit). The circuits were based on bacterial DNA-binding repressor proteins and corresponding DNA operator sequences. Since then the field of genetic circuit design has been rapidly advancing [reviewed in (Xia et al., 2019)]. Transcriptional regulatory circuits based on libraries of orthogonal repressor-operator pairs (Nielsen et al., 2016), RNA interference (Bloom et al., 2014), and more recently CRISPR-dCas complexes (see below) have been constructed. The design of complex multi-layered genetic circuits usually employs abstract principles similar to those used in the construction of digital electronic circuits from elementary modules, called logic gates. These logic gates (such as AND, OR, NOT, etc.) produce discrete output signals depending on one or more input signals (in genetic circuits rates of specific gene expression can be treated as such signals). Practical applications of genetic circuits include the design of living therapeutics (Brennan, 2022) [including those for altering the human microbiome (Robinson et al., 2022) or designing advanced CAR T therapies for cancer treatment (Cho et al., 2018a)], biosensors (Del Valle et al., 2021; Nguyen et al., 2021), tissue engineering in regenerative medicine (Healy and Deans, 2019), genetic modification of crop plants (de Lange et al., 2018), etc.

The discovery of CRISPR-Cas adaptive immunity systems in bacteria and archaea (Makarova et al., 2020) and the demonstration of their application for targeted genome editing in bacteria and eukaryotes (Jinek et al., 2012; Cong et al., 2013) gave impetus to a whole new era in synthetic biology. Soon afterward it was demonstrated that catalytically dead CRISPR-Cas9 proteins can be repurposed to interfere with gene transcription (Qi et al., 2013) and used as vehicles to deliver fused moieties to the specific genome loci in a programmable manner (Xu and Qi, 2019). These discoveries spurred rapid progress in the application of CRISPR-dCas technology for the construction of transcriptional genetic circuits in recent years. In this mini-review we survey the diversity and properties of CRISPR-dCas-based systems, analyze reported and prospective applications thereof for genetic circuits design, outline challenges and further research directions in the field.

## 2 The properties and diversity of CRISPR-dCas systems for genetic circuit design

CRISPR-dCas systems are derived from wild-type CRISPR-Cas systems by inactivating their nuclease activity *via* point mutations while retaining their ability to bind DNA loci. In nature, CRISPR-Cas systems are represented by a very diverse group of genes (Makarova et al., 2020). For practical applications, class 2 type II (based on Cas9 effector nucleases) and type V (based on a heterogeneous family of Cas12 nucleases) are most valuable since their effector nucleases are mainly DNA binding and are represented by a single protein.

**FIGURE 1:**
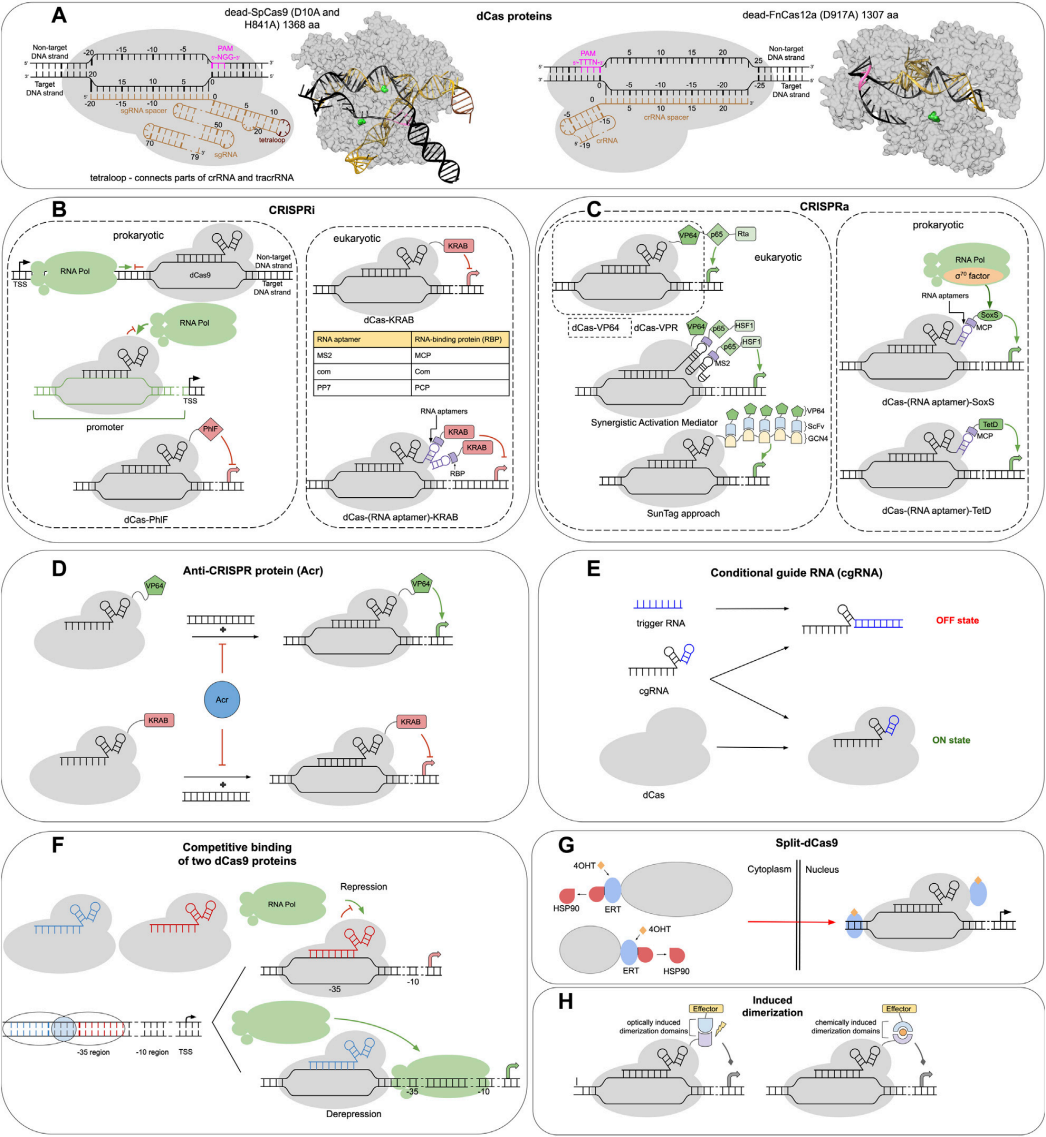
CRISPR-dCas toolbox for genetic circuit design. **(A)** Schematic and structural representation of the dCas proteins: dead-SpCas9 (left) and dead-FnCas12a (right). The colors of elements in the annotated schematic representations correspond to those in the structural representation. Amino-acid residues which are mutated in the dCas proteins are highlighted in green, PAM stands for protospacer adjacent motif. **(B–H)** dCas-based constructs that can be used for transcriptional gene expression control. **(B)** Principal schema of sequence-specific gene repression using CRISPR interference (CRISPRi) in prokaryotes (left) and eukaryotes (right). **(C)** Various approaches to sequence-specific gene activation using CRISPR-dCas systems (CRISPRa) in prokaryotes (right) and eukaryotes (left). **(D)** Additional regulation of CRISPRi and CRISPRa by Anti-CRISPR proteins (Acr). **(E)** Regulation of dCas activity *via* conditional sgRNA and trigger RNA. **(F)** Combination of two interfering CRISPR-dCas complexes for fine control of target gene expression. **(G)** Activation of split-dCas9 proteins fused to ERT domains of estrogen receptor (see text). **(H)** Additional control of sequence-specific dCas targeting activity for gene expression regulation by chemical- or light-induced dimerization domains.

The most studied Cas9 nuclease is SpCas9 from *S. pyogenes*, a 158 kDa (1368 aa) protein that in nature forms a complex with two RNA molecules—CRISPR RNA and transactivating CRISPR RNA. The former encodes the spacer sequence that is used to guide target-specific SpCas9 binding. Key elements of both RNAs may be combined *via* a tetraloop into a single guide RNA (sgRNA), which binds to SpCas9 with a Kd of around 10 pM (Wright et al., 2015) (see Figure 1A). The process of SpCas9-sgRNA binding to the DNA is now known in detail (Wang et al., 2022). As a first step, the SpCas9-sgRNA complex finds a three-nucleotide-long protospacer adjacent motif (PAM) on the non-target DNA strand *via* its C-terminal domain (NGG is the optimal PAM sequence for SpCas9). Next, the “recognition” and “nuclease” lobes of Cas9 rotate, simultaneously bending the DNA and exposing the PAM-proximal bases on the target DNA strand for sgRNA invasion and further target sequence interrogation through base pairing, resulting in the formation of an R-loop structure. The formation of a full RNA-DNA duplex and its conformational kinking activates the nuclease domains (RuvC and HNH) which cleave the DNA strands. Two mutations (D10A, H841A) in SpCas9 inactivate these domains resulting in a dead-SpCas9 (SpdCas9).

The use of SpdCas9 to interfere with RNA polymerase (RNAP) binding or progression along the DNA in prokaryotes resulted in the first application of such systems for sequence-specific control of gene expression (termed CRISPRi) (see Figure 1B) (Qi et al., 2013). Qi et al. (2013) have shown that up to 300-fold gene expression repression can be achieved by targeting dCas9 to the gene promoter region (blocking RNAP binding) or the beginning of the gene coding sequence (interfering with transcription elongation). Interestingly, in the latter case, the effect is only achieved when targeting the DNA non-template strand (Qi et al., 2013). The specificity and strength of the dCas9-sgRNA complex binding to the target DNA sequence is governed by around 20 nucleotides at the 5′-end of sgRNA—the so-called spacer sequence. The 7–12 bp long PAM proximal part of the spacer (usually called the seed sequence) is especially important for binding specificity. Mismatches in this region have a severe impact on binding or cleavage, while the PAM distal region can tolerate multiple mismatches (Feng et al., 2021). Seed sequence mismatches result in a 70%–90% decrease in gene repression in CRISPRi assays (Qi et al., 2013). The overall Kd for Cas9–RNA complex binding to a *bona fide* DNA target has been estimated to be around 0.5 nM (Sternberg et al., 2014). For comparison, the high-affinity bacterial transcription repressor LacI binds to its cognate operator LacO *in vitro* with a Kd of around 10 pM (Du et al., 2019), while eukaryotic p53 dimers bind to their response elements with Kds above 5 nM (Weinberg et al., 2005).

A family of dCas9 proteins may be derived from SpCas9 orthologs in various organisms. These Cas9 proteins may vary in size (e.g., 984 aa in *C. jejuni*, 1629 aa in *F. novicida*), recognized PAM sequences (e.g., NNGRRT in *S. aureus*, NNNNGATT in *N. meningitidis*, NNARAAW in *S. thermophilus*), optimal spacer length (e.g., 21–24 nt in *N. meningitidis*), the structure of CRISPR RNA (Anzalone et al., 2020). So far dCas9 from *S. thermophilus*, *N. meningitidis*, *T. denticola* have been used (Brocken et al., 2018). An additional advantage of having different dCas9 orthologs at hand is the simultaneous use of several orthogonal dCas9 (i.e., binding specifically only their cognate CRISPR/sgRNAs) (Esvelt et al., 2013). For a multi-layered gene circuit (see below) this may alleviate competition between several sgRNAs for the same pool of dCas9 protein, potentially increasing circuit robustness and alleviating interference between genetic components.

Cas12 nucleases are another source of CRISPR-dCas systems. The Cas12 family is more diverse than Cas9 family and is further classified into different subtypes (e.g., Cas12a from *F. novicida*, *E. eligens*, *Acidaminococcus* sp., *Lachnospiraceae* sp. or Cas12e from Deltaproteobacteria) (Makarova et al., 2020). Cas12 nucleases usually require an AT-rich PAM site located upstream of the target region (contrary to Cas9), many lack tracrRNA and have only one nuclease domain (RuvC) which can be inactivated by a point mutation to produce dCas12 (Anzalone et al., 2020) (Figure 1A). One advantage of certain dCas12a variants is their nuclease-independent ability to process pre-crRNA into crRNA, thus many crRNAs may be simultaneously encoded by one customized CRISPR array even for CRIPSRi applications (Zetsche et al., 2017). In prokaryotes, initial dCas12a-based CRISPRi assays have demonstrated up to ∼8-fold gene expression repression (Kim et al., 2017). Interestingly, unlike dCas9, dCas12a has been shown to repress transcription elongation much better when targeted to the template DNA strand rather than the coding strand (Kim et al., 2017). Disadvantages of wild-type dCas12a versus dCas9 include a more restrictive PAM (e.g., TTTV for AsCas12a) and lower regulation efficiency, although engineered dCas12a have improved properties (see below) (Kim et al., 2017).

During the last decade many engineered Cas9, Cas12 variants, and sgRNAs have been developed for genome editing, aiming to relax PAM requirements (Collias and Beisel, 2021), reduce off-target activity, and increase on-target activity (Anzalone et al., 2020; Riesenberg et al., 2022). Simultaneously optimizing all these properties is difficult, since the relaxation of PAM requirements or reducing off-target activity often comes at a cost of the reduced on-target activity. For the purpose of dCas applications, what essentially matters is the on-target and off-target binding of dCas, which may not be directly related to the nuclease activity of its catalytically active counterpart. Since the high concentration of dCas proteins is usually required for the effective operation of genetic circuits, dCas toxicity [likely caused by its off-target binding, which may happen even without sgRNA (Jones et al., 2017)] poses an important problem, especially in bacteria (Zhang and Voigt, 2018). An original approach to alleviating dCas9 toxicity suggested by Zhang and Voigt involved reducing dCas DNA affinity by mutating its PAM-recognizing domain and simultaneously fusing it to another sequence-specific transcription factor to rescue DNA specificity and affinity of the whole construct (Zhang and Voigt, 2018) (see Figure 1B, bottom). Such a design allowed to reach ∼9,000 dCas9 molecules per *E. coli* cell compared to ∼500 in the wild-type case. Some other examples of dCas engineering include altering the PAM interacting domain of SpdCas9 to specifically recognize start codons of protein-coding genes (5′-CAT-3′ PAM) (Wang et al., 2021) and development of hyperdCas12a—a version of dCas12a from Lachnospiraceae bacterium with enhanced binding affinity resulting in significantly improved CRISPRi and CRISPRa outcomes in eukaryotes (Guo et al., 2022).

## 3 Enhancing dCas-systems *via* fusion domains and/or intermolecular interactions

The versatility of dCas-systems in gene expression regulation can be significantly enhanced by combining them with other functional protein domains. For instance, in bacteria instead of transcription repression, gene activation can be achieved by fusing dCas with prokaryotic activator domains and targeting them near promoters (CRISPRa technology) (Figure 1C). A number of such activator domains have been used so far, including the omega subunit of RNAP, TetD, and SoxS activators from *E. coli* (Dong et al., 2018; Villegas Kcam et al., 2021). SoxS-based designs have been found to confer the best results, yielding up to a 50-fold increase in mRNA levels of activated genes, provided the SoxS domain has proper positioning and orientation relative to the promoter (Dong et al., 2018; Klanschnig et al., 2022).

In eukaryotes, the effects of bare dCas-sgRNA binding on transcription repression are limited (around two-fold repression has been reported for human cells) (Qi et al., 2013). This is likely due to the much more complex mechanisms of transcription regulation in chromatin, where nucleosomes alone may block transcription elongation unless other chromatin factors are recruited to help (Bondarenko et al., 2006; Petesch and Lis, 2012). Using dCas as a vehicle to target transcriptional effectors is a way to address this challenge and expand the toolbox toward gene activation (Figures 1B, C). To enable gene silencing in eukaryotes, linking several chromatin effectors to dCas9 proteins has been attempted [see (Gilbert et al., 2013; Xu and Qi, 2019)]. For human cells, the most efficient results (up to 15-fold repression of GFP expression in HEK-293 cells), so far, were achieved by fusing dCas9 to the KRAB domain (Krüppel associated box) of Kox1 protein (Gilbert et al., 2013; Gilbert et al., 2014), which was successfully used in the construction of complex genetic logic gates (Kim et al., 2019). KRAB interacts with KAP1 protein, which acts as a scaffold for various heterochromatin-inducing factors (HP1, SETDB1, NuRD, N-CoR1, еtc.) (Groner et al., 2010). In yeast, fusing dCas9 with Mxi1 domain, which is thought to interact with the histone deacetylase Sin3, yielded the best results in maintaining consistent gene repression states (more than 10-fold repression of GFP expression was achieved) (Gilbert et al., 2013; Gander et al., 2017).

For CRISPR-mediated activation in eukaryotes (CRISPRa) initial works relied on a VP64 transcription activator derived from the herpes simplex virus. Although moderately effective [25-fold activation reported by Gilbert et al. (2013) in human HEK293 cells], it has been successfully used in the construction of genetic circuits and AND gates in yeast (Hofmann et al., 2019). Further CRISPRa studies focused on developing more potent and more flexible activator designs. Chavez et al. (2015) found that adding a tripartite VPR activator consisting of serially connected VP64, p65, and Rta domains to the C-terminus of dCas9 resulted in more than an order of magnitude better activation than in the dCas9-VP64 case (up to a 320-fold increase in activation) (see Figure 1C). The dCas9-VPR was employed for the construction of AND gates in human HEK293T cells (Gao et al., 2016). Another highly potent but somewhat more complex CRISPRa design is the synergistic activation mediator (SAM), yielding more than 100-fold greater activation than dCas9-VP64. SAM combines dCas9-VP64 protein with a special scaffold sgRNA which incorporates two MS2 RNA hairpin aptamers that recruit exogenously introduced MCP-p650-HSF1 constructs *via* MS2 binding to the bacteriophage MS2 coat protein (MCP) (Konermann et al., 2015). A more detailed discussion of the effectiveness of different CRISPRa methods in eukaryotes can be found elsewhere (Shakirova et al., 2020).

The use of RNA hairpins and their corresponding protein binding domains provides a versatile platform for the modular design of dCas-based systems. Using this strategy Zalatan et al. (2015) have simultaneously implemented CRISPRa together with CRISPRi using one dCas9 protein but different sgRNAs attached to various aptamers (Figures 1B, C). Interestingly, the recruitment of VP64 domain through an MS2-MCP interaction yielded ∼18-fold better gene activation results than in the case of fusing VP64 directly to the dCas9 protein. This can be in part explained by the fact that MCP binds as a dimer to its RNA target. Such RNA-mediated scaffolding designs are not without limitations, since RNA hairpins interfere with sgRNA transcription. Routes to circumvent this problem include using Pumilio RNA-binding protein that binds to a non-hairpin structure (Cheng et al., 2016) or coupling activators *via* antigen-antibody interactions derived system [e.g., SunTag system (Tanenbaum et al., 2014)].

The interaction of dCas-RNA complexes with regulatory proteins or RNAs provides additional instruments to the toolbox (Figures 1D, E). AntiCRISPR proteins (Acrs), that bind and inactivate dCas were shown to practically abolish CRISPRa and CRISPRi activity of dCas-based systems. Arcs have been used in genetic circuits to construct feed-forward loops and pulse generators (Nakamura et al., 2019). An alternative way to deactivate dCas complexes is to inactivate or activate sgRNA. To this end, conditional guide RNAs (cgRNA) have been designed. They can be blocked or activated by binding a trigger RNA. The effectiveness of such activation/deactivation of up to 50% has been achieved (Hochrein et al., 2021). cgRNA may be used as a signal input in genetic logic gates (Hochrein et al., 2021). Alternatively, aptazyme, which upon binding of a small ligand (guanine), catalyzes self-cleavage and consecutive activation of sgRNA was used as a way to trigger CRISPRa. However, such a design had significant levels of gene activation even without guanine (Tang et al., 2017). Finally, competition of two dCas-sgRNA complexes that target neighboring DNA sites has been used to expand the flexibility of dCas-based circuits (Anderson and Voigt, 2021) (Figure 1F). Such a design is good for ratiometric signal processing when the relative strength of two signals is used as an input to the circuit.

Another powerful strategy used to expand dCas-based instruments’ repertoire is the use of split proteins, including those with induced dimerization and split-dCas itself (Figures 1G, H). Chemically induced dimerizers (CID), such as FKBP/FRB system induced by rapamycin (Gao et al., 2016; Bao et al., 2017), or optogenetically induced dimerizers (OID), such as the Magnet system (Kawano et al., 2015; Nihongaki et al., 2017) have been used to link dCas to transcriptional effectors. Such designs provide spatiotemporal control of gene expression, have almost no background activity when no dimerizing agent is present, and manifest efficiencies comparable to direct dCas protein fusion constructs [e.g., up to 165-fold activation in CRISPRa using VPR (Gao et al., 2016)]. Finally, the application of split-dCas proteins has been reported. In one case split-dCas domains of a dCas9-VP64 system were fused to the CID-domains of the FKBP/FRB system (Zetsche et al., 2015). However, such a design was shown to have a considerable level of background activation due to the spontaneous auto-assembly of dCas9 *in vivo* (Zetsche et al., 2015). To dampen auto-assembly, split-dCas parts have to be kept further apart. In eukaryotes, this was shown to be possible through the differential addition of nuclear localization and nuclear export signals to the split parts (Zetsche et al., 2015). Alternatively, fusing dCas9-split parts to ligand-binding domains from nuclear receptors was demonstrated to be effective. Nguyen et al. (2016) have fused dCas9 split parts to the ERT domains of the estrogen receptor. ERT keeps dCas in the cytoplasm due to interactions with Hsp90, unless 4-hydroxytamoxifen is added. 4-hydroxytamoxifen disrupts the interactions and allows dCas parts to enter the nucleus (Figure 1G). In the nucleus, they complement each other to form a functional complex. Such systems were shown to induce ∼100-fold gene activation, which was ∼2.5-fold less than achievable with non-split versions of dCas9-VPR systems (Nguyen et al., 2016).

## 4 Logic gates and multi-layered CRISPR-based genetic circuits

**FIGURE 2:**
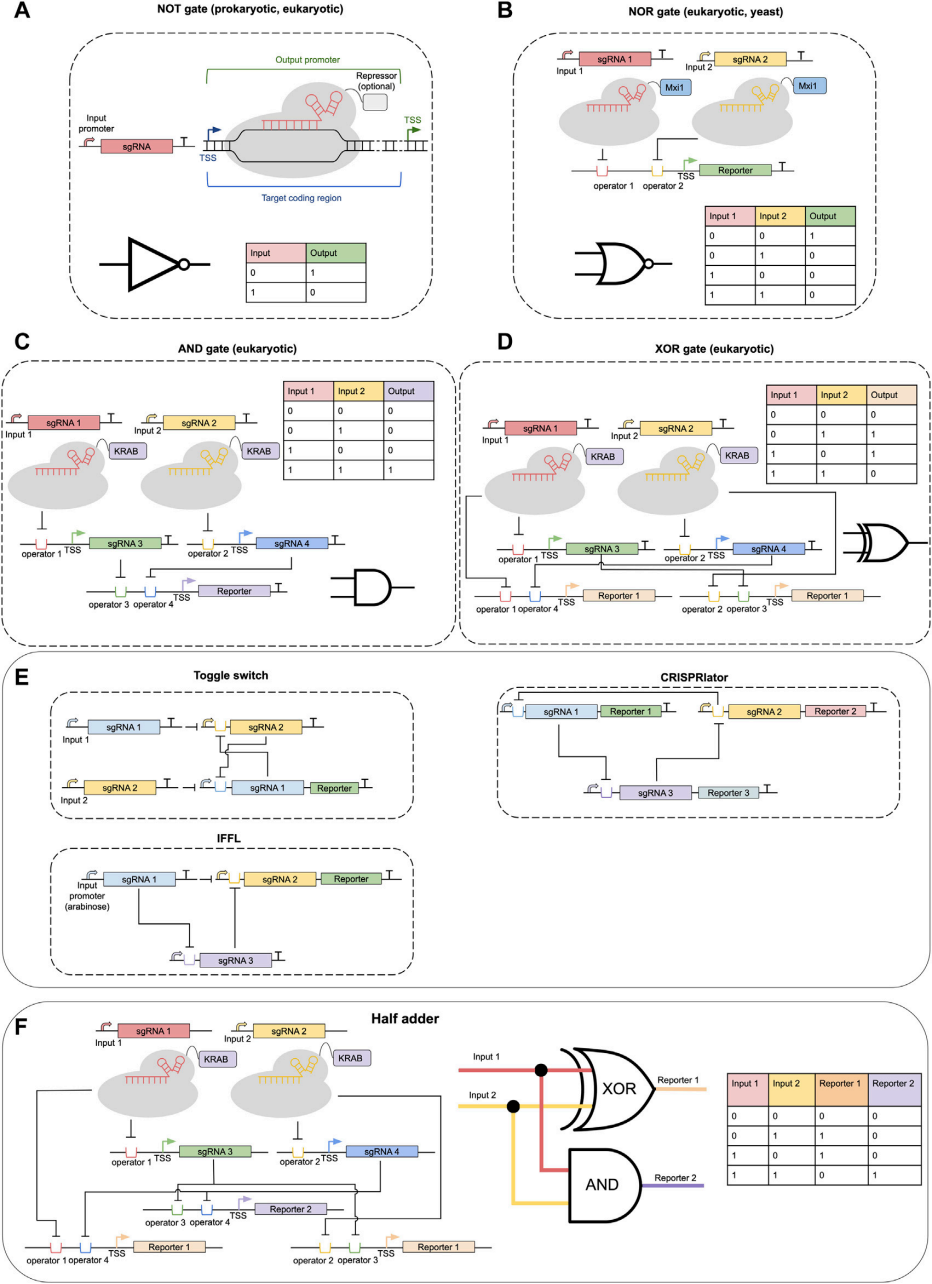
CRISPR-dCas-based genetic circuits. **(A)** A NOT gate based on CRISPRi approach. **(B)** A typical NOR gate in eukaryotes built with dCas9 fused to a Mxi1 chromatin repressor domain. **(C)** A variant of AND gate based on the CRISPRi approach. **(D)** A variant of XOR gate based on the CRISPRi approach. **(E)** Simple genetic circuits: toggle switch, incoherent feed-forward loop, CRISPRlator. **(F)** A two-layer CRISPR-dCas-based circuit implementing a half-adder device. Where appropriate truth tables and schematic representations of logic gates are shown.

Borrowed from the world of digital electronics, logic gates are a key abstraction used to describe and design genetic circuits (see Figure 2). In transcriptional genetic circuits “signals” are rates of transcription initiated at specific promoters. It is simple to see that CRISPRi technology can be straightforwardly used to implement a transcriptional inverter, also called a NOT gate. In this case transcription of sgRNA (controlled by an input promoter) results in the repression of an output gene (Figure 2A) (Didovyk et al., 2016; Tickman et al., 2022). For practical applications, however, the exact quantitative characteristics of NOT gates have to be taken into account (ON/OFF-state expression levels, transfer function shape and dynamic range).

Of more practical interest is the creation of gates that have two inputs (AND, OR, AND NOT = NAND, OR NOT = NOR, etc.), since they can be combined to create logic devices of arbitrary complexity. In *E. coli*, the first NOR gates were implemented by Nielsen and Voigt (2014). In their design, two different input promoters regulate the transcription of identical sgRNAs, which in turn target dCas9 to a single DNA locus near the promoter of an output reporter gene. In eukaryotes, when using dCas-fusions with regulatory domains, greater flexibility is allowed in terms of the exact positions that may be targeted by dCas for gene repression or activation. This allows the use of two different sgRNAs as input signals, which target dCas constructs to the two operator sequences near the promoter of an output gene (see Figure 2B). Such strategies have been successfully implemented in yeast and human HEK-193T cells (Gander et al., 2017; Kim et al., 2019). Potentially, it should be possible to create an OR gate using the CRISPRa approach in a similar way, however, it has not yet been reported in the literature to our knowledge. AND and XOR gates may be implemented as a two-layer circuit by combining NOR and two NOT gates (Figures 2C, D) (Kim et al., 2019). Design of NAND/AND gates is also possible by considering dCas expression itself as one of the transcriptional input signals (Liu et al., 2014; Hofmann et al., 2019), however, such gates are hard to use as modules in multi-layer circuits. Alternatively, a conditional sgRNA approach may be employed to create AND gates driven by only RNA input signals: The conditional sgRNA and trigger RNA that activates it (Hanewich-Hollatz et al., 2019).

One of the promises of CRISPR-dCas technology is the assumed ease to create multi-layer circuits driven by sets of orthogonal sgRNA, where the expression of one specific sgRNA regulates the expression of other sgRNA and so on (see, example circuits in Figures 2E, F). Indeed, such layering has been shown to be feasible. For example, Tickman et al. (2022) have combined CRISPRi and CRISPRa in *E.coli* to make three-layer systems, including feed-forward loops (FFL). Gander et al. (2017) have constructed genetic circuits in yeast based on NOR gates with up to seven layers. Santos-Moreno et al. (2020) were able recently to implement classical genetic circuits such as a bistable toggle switch and repressilator (termed CRISPRlator) in *E. coli* using CRISPRi (Figure 1E). And Kim et al. (2019) have developed a platform based on dCas9-KRAB and four sgRNAs that allowed the realization of various one and two-layer circuits in HEK293T mammalian cells, including a half-adder circuit (Figure 2F).

## 5 Challenges of CRISPR-based gene circuits

Implementation of multi-layered gene circuits is not without challenges. Some of the key requirements for an effective operation of a multi-layered genetic circuit are the orthogonality of its modules (low crosstalk between signals), compatibility of input and output signals between modules (signal thresholds and dynamic ranges, acceptable levels of transcriptional leak, shape, and quality of signal transfer functions), the robustness of genetic elements functioning with respect to their genetic context (Brophy and Voigt, 2014).

For example, the choice of orthogonal sgRNA libraries and their operator targets is not a straightforward task, although some algorithms have been suggested (Didovyk et al., 2016). An optimally designed sgRNA library should at least take into account 1) potential crosstalk due to dCas-sgRNA tolerance to base pairing mismatches especially in the 5′-end of the spacer and 2) potential off-target binding to the genomic DNA. Moreover, the effectiveness of sgRNA transcription from the respective genetic constructs and its post-transcriptional processing have to be taken into account. In prokaryotic systems, one challenge is transcriptional read through due to the short nature of sgRNAs. To mitigate this problem strong terminators should be used (Nielsen and Voigt, 2014). In eukaryotes, nuclear export signals, the 5′-cap and 3′-poly-A tail, interfere with sgRNA functioning. To mitigate these effects flanking sequences are often introduced into the constructs of sgRNAs that are cleaved after transcription. Cleavage has been achieved through ribozymes (Gander et al., 2017) or the tRNA cleavage system (Kim et al., 2019).

Context-dependent effects, which alter operator performance and gene expression (especially if promoter sequence is altered) are another problem for modular circuit design. These effects may be due to the influence of nearby sequences on promoter melting, RNA hairpins that block ribosome binding sites, or transcriptional read-through (Brophy and Voigt, 2014). In eukaryotes, the influence of chromatin structure and dynamics adds another layer of complexity. In genome-wide screens, it was found that both the dCas9-Mxi1 and dCas9-KRAB constructs strongly suppress transcription when targeted to some regions of DNA, but not others (Gilbert et al., 2013). Nucleosomes are known to impede Cas9 access to the DNA (Horlbeck et al., 2016), so their positioning might be another factor that has to be taken into account.

One other challenge of CRISPR-dCas systems is their toxicity, especially in bacteria (Cho et al., 2018b), likely originating from dCas non-specific binding. Certain engineered dCas proteins may be used to address this problem (see above) (Zhang and Voigt, 2018). We hypothesize that the use of dCas-proteins with longer and more specific PAM sequences may actually be beneficial for genetic circuit design. It might reduce toxicity by lowering off-target binding, while specific PAMs may be easily incorporated into the genetic elements of the circuit, which are anyway designed artificially.

Optimization of dose-response transfer functions and signal thresholds is another challenge. The transfer functions of dCas-based genetic elements depend on many factors, including the concentration of dCas protein, sgRNA and their target DNA site, their on-target and off-target binding efficiencies, interactions of fused effector domains with transcription and chromatin machinery, etc. In the design process, plasmid copy numbers and expression levels of individual components of the gene circuit should be carefully optimized (Nielsen and Voigt, 2014). One drawback of dCas-based transfer functions is their linear-like dose-response characteristics. In conventional transcription genetic circuits, based on repressor proteins, digital-like S-shaped dose-response curves are obtained due to cooperative DNA binding (due to repressor dimerization or DNA looping) (Brophy and Voigt, 2014). dCas-sgRNA complexes *per se* do not bind cooperatively to the DNA, however, additional factors, such as fusion of chromatin effector domains or depletion of dCas pool due to non-specific binding of genomic DNA (Santos-Moreno et al., 2020) may change the dose-response curve in a favorable direction. This is, for instance, needed for the design of bistable toggle switch circuits (Santos-Moreno et al., 2020).

## 6 Discussion

Despite certain challenges, CRISPR-dCas-based tools offer great flexibility in designing multi-layer synthetic genetic circuits. Their versatility already allowed to create rather complex circuits in bacterial (Tickman et al., 2022), mammalian (Kim et al., 2019), yeast (Gander et al., 2017), and even plant cells (Khan et al., 2022). Such terms as “genetic programs,” “genetic computation,” “complex logic computation” and even “CRISPR/Cas9-based core processor” are now being used to refer to such circuits. We envision that further progress in optimizing and characterizing CRISPR-dCas components should be bridged with attempts at automated *in silico* design of such circuits with sufficient confidence. Practical applications of dCas-based genetic circuits would require connecting such circuits to effective signal input and output molecular devices. Such technologies are already being developed. For example, promising results have been already obtained in the activation of dCas by soluble extracellular factors using fusion constructs between antibodies, dCas-proteins, and proteases (Schwarz et al., 2017). Taken together, dCas-based genetic circuits are a promising technology that will likely contribute to the development of new cell-based therapeutics, diagnostic devices, sensors, epigenetic therapies, tissue engineering, and development of plants with improved properties and serve as a tool to understand genome regulation in living organisms.
